# Population pharmacokinetic model of cefazolin in total hip arthroplasty

**DOI:** 10.1038/s41598-021-99162-7

**Published:** 2021-10-05

**Authors:** J. Lanoiselée, R. Chaux, S. Hodin, S. Bourayou, A. Gibert, R. Philippot, S. Molliex, P. J. Zufferey, X. Delavenne, E. Ollier

**Affiliations:** 1 Dysfonction Vasculaire et Hémostase, INSERM, U1059, 42023 Saint-Étienne, France; 2 Département d’Anesthésie-Réanimation, CHU de Saint-Etienne, 42055 Saint-Étienne, France; 3 Unité de Recherche Clinique Innovation et Pharmacologie, CHU de Saint-Etienne, 42055 Saint-Étienne, France; 4 Service de Chirurgie Orthopédique et Traumatologique, CHU de Saint-Etienne, 42055 Saint-Étienne, France; 5 Laboratoire de Pharmacologie Toxicologie, CHU de Saint-Etienne, 42055 Saint-Étienne, France

**Keywords:** Pharmacokinetics, Antibiotics

## Abstract

Cefazolin is an antibiotic recommended for infection prevention in total hip arthroplasty (THA). However, the dosing regimen necessary to achieve therapeutic concentrations in obese patients remains unclear. The aim of this study was to conduct a population analysis of cefazolin pharmacokinetics (PK) and assess whether cefazolin administration should be weight adapted in THA. Adult patients undergoing THA surgery received an injection of 2000 mg of cefazolin, doubled in the case of BMI > 35 kg/m^2^ and total body weight > 100 kg. A population PK study was conducted to quantify cefazolin exposure over time compared to the therapeutic concentration threshold. A total of 484 cefazolin measurements were acquired in 100 patients, of whom 29% were obese. A 2-compartment model best fitted the data, and creatinine clearance determined interpatient variability in elimination clearance. Our PK simulations using a 2000 mg cefazolin bolus showed that cefazolin concentrations remained above the threshold throughout surgery, regardless of weight or renal function. A 2000 mg cefazolin single injection without adaptation to weight or renal function and without intraoperative reinjection was efficient in maintaining therapeutic concentrations throughout surgery. The optimal target concentration and necessary duration of its maintenance remain unclear.

## Introduction

Postoperative surgical site infections (SSIs) are a common complication following total hip arthroplasty (THA) surgery. Deep prosthetic joint infections (PJIs) occur with an incidence ranging from 0.1 to 11.1%^1^ and are associated with poor outcomes in terms of in-hospital length of stay, rehospitalization and reoperation^2^. They result in an altered quality of life for the patients^3^ and increased medical costs for the healthcare system^3^.

Antimicrobial prophylaxis is the most effective strategy to prevent PJIs^4^. Cefazolin, a first-generation cephalosporin, is currently administered for routine perioperative use to every patient undergoing THA. It possesses good properties in terms of its bioavailability, cost-effectiveness and tissue distribution^5–7^. Its broad spectrum of antimicrobial activity covers the most common pathogens responsible for SSI, namely, coagulase-negative Staphylococci (CoNS) and *Staphylococcus aureus*, which contribute to 50–60% of PJIs^8^.

The maximum effect for effective prophylaxis with cefazolin is obtained when serum concentrations exceed the minimum inhibitory concentration (MIC) against the target bacteria throughout surgery^9^. However, the dosing regimen necessary to achieve such concentrations remains unclear. Creatinine clearance seems to influence cefazolin concentrations over time^10^. However, it is unknown whether an adaptation to body weight could optimize cefazolin exposure during surgery.

Current guidelines regarding perioperative antibiotic prophylaxis in THA recommend a dose adjustment to patient weight^11–13^, but other recommendations did not address this issue^14^. From a pharmacokinetic (PK) perspective, this strategy lacks scientific justification^15^. The results of studies regarding cefazolin concentrations after prophylactic administration in obese patients are heterogeneous. Guidelines recommending an increased cefazolin dosing in obese patients were determined on the basis of small studies with patients undergoing bariatric surgery or caesarean section ^16^. Obese patients receiving a 2 g injection did not develop a significantly higher SSI rate compared with non-obese patients, but there were trends toward an increase, justifying the recommendation considering the low cost and favourable safety profile of cefazolin. One PK modelling study evaluated cefazolin exposure in THA surgery, but the authors of this work did not include obese patients in their study population^10^.

The aim of this study was to conduct a population analysis of cefazolin PK in adult patients undergoing THA to quantify the factors responsible for cefazolin PK variability, evaluate their effect on drug exposure and assess whether cefazolin administration could be optimized in obese patients undergoing THA surgery.

## Materials and methods

We performed a PK study based on data from a randomized trial in THA comparing two regimens of tranexamic acid (TXA) administration with a PK/PD analysis. Residual blood sampled from the TXA study was used to perform the cefazolin PK analysis^17^.

### Study design

The PeriOpeRative Tranexamic acid in hip arthrOplasty (PORTO) study was conducted in accordance with the ethical principles stated in the Declaration of Helsinki, Good Clinical Practices and relevant French regulations regarding ethics and data protection. The protocol and amendments were approved by the relevant central independent ethics committee (Comité de Protection des Personnes Sud-Est1). This study is registered at the French National Agency for Medicines and Health Products Safety (ANSM; ref: 130908A-21), EudraCT (ref: 2013-000791-15) and ClinicalTrials.gov (no. NCT02252497).

Consecutive patients aged over 18 years undergoing cementless primary unilateral THA surgery at the University Hospital of Saint Etienne, France, were enrolled for inclusion between April 2014 and December 2015. Subjects were included in the study if they were scheduled to receive cefazolin for antibiotic prophylaxis.

The choice of anaesthesia was left to the discretion of the anaesthetists. Cefazolin was administered according to the French guidelines for antibiotic prophylaxis in patients undergoing THA^18^. Patients received a direct intravenous bolus injection of 2000 mg of cefazolin at anaesthesia induction, with a 1000 mg redosing in case of surgery duration longer than 4 h. This dose was doubled in cases of BMI > 35 kg/m^2^ and total body weight > 100 kg. All patients were operated on in a lateral position through a posterior approach.

### Sample collection and drug assay

Venous blood samples were drawn into lithium heparin-coated tubes at the following times: 3 min and 20 min after the bolus of cefazolin, at the end of surgery, and 3 h and 8 h after the bolus of cefazolin.

The samples were stored at -80 °C until analysis. Total plasma cefazolin concentrations were measured using an Ultimate U3000 liquid chromatography system (Thermo Fisher Scientific) coupled with a Q-Exactive Plus mass spectrometer (Thermo Fisher Scientific).

The analyses were performed in positive ionization in parallel reaction monitoring mode at a resolution of 35 000 (at m/z 200) both for cefazolin in plasma samples (mass/charge [m/z] 455.03732) and for the internal standard ([^13^C_2_,^15^N]-cefazolin; m/z 458.04089). A target ion (for quantification) and a confirming ion were monitored. For cefazolin and its IS, the target ions were m/z 323.05563 and 326.0593, confirming that the ions were m/z 295.06079 and m/z 298.06424, respectively.

The mobile phase was a mixture of (A) 0.1% formic acid in water and (B) 0.1% formic acid in acetonitrile, applied as a gradient to a Hypersild Gold C18 column (50 mm × 2.1 mm, 3 μm) (Thermo Fisher Scientific). Samples were prepared by dilution of 50 µL of the plasma sample with 500 µL of methanol. After centrifugation, 50 µL of supernatant was diluted in 1 mL of IS in water. The method was linear over the concentration range of 5–500 mg/L. The lower limit of quantification was 5 mg/L. The inter- and intraday precisions were evaluated at four quality control levels, and the coefficients of variation were under 10%.

### Model development and evaluation

Data were analysed using MONOLIX modelling software (version 4.3, release 3, Lixoft) as previously described^19,20^. Cefazolin concentrations were analysed using the following nonlinear mixed-effect model framework:

$$Obs_{ij} = F\left( {t_{ij} ,\phi_{i} } \right) \, + \left( {a + b \times F\left( {t_{ij} ,\phi_{i} } \right)} \right) \times \varepsilon_{ij}$$

where *Obs*_*ij*_ denotes the observed concentration measured for patient i at time j; the function *F*(*t*_*ij*_,*ϕ*_*i*_) corresponds to the concentration predicted by the model for patient i at time j with individual PK parameters *ϕ*, and parameters a and b being the constant and proportional components, respectively, of the error model with *ε*_*ij*_ ~ N (0, 1).

We used the stochastic approximation expectation maximization algorithm to estimate the maximum likelihood of the model^21^. The model was built according to a stepwise procedure, with initial identification of the best structural model for cefazolin PK without covariates, by estimating 1-, 2-, and 3-compartment PK models.

We then evaluated the effect of covariates on cefazolin exposure. The covariates tested included age, various body size descriptors such as total body weight (TBW), body mass index (BMI) and lean body weight (LBW), creatinine clearance (CrCL) according to the Cockcroft-Gault (C&G) formula^22^ and the Chronic Kidney Disease–Epidemiology Collaboration (CKD-EPI) formula^23^, and sex. Covariates were tested using a stepwise procedure and were kept in the model if they improved the goodness of fit, reduced interindividual variability and decreased the Bayesian Information Criteria (BIC) in comparison to the previous model. Covariates were tested with allometric scaling according to the following equation [using clearance (CL) as an example]:

$$CL_{i} = CL_{POP} \times \left( {\frac{{CrCL_{i} }}{80}} \right)^{{\theta_{CrCL} }} \times e^{{\eta_{i} }}$$

where $CL_{i}$ and $CrCL_{i}$ are the individual values of CL and CrCL, respectively, for patient i, $CL_{POP}$ is the estimated typical population value of CL, $\theta_{CrCL}$ is the estimated effect factor for CrCL, and $\eta_{i}$ is the random effect for patient i, assumed to be normally distributed with a mean of zero and a variance equal to $\omega_{CL}^{2}$ (N[0,$\omega_{CL}^{2}$]). $CrCL_{i}$ is centred on 80 to provide an estimation of CL for the typical CrCL value in our population.

Model evaluation was based on inspection of the prediction-corrected visual predictive check (pred-corrected VPC)^24^ and the goodness of fit, using the dataset used to build the model. The pred-corrected VPC was obtained by generating 1000 simulations of the subject’s PK parameters using the final model. The capacity of the model to predict the observed data was evaluated by comparing the distribution of the simulated concentrations with the observations. The goodness of fit was determined by plotting the observed values versus the population predictions of the model, the normalized prediction distribution errors (NPDE) versus time and the NPDE versus predictions^25^. Selection of covariates was performed using a forward stepwise algorithm using the Bayesian information criterion (BIC), the model with the lowest BIC being the best model to describe the data^26^.

### PK simulations

Finally, we generated PK simulations using our population model to characterize the effect of significant covariates on cefazolin PK, evaluate cefazolin exposure over time compared to the therapeutic concentration threshold, and assess whether a dose adjustment to body weight according to the French guidelines could optimize cefazolin exposure in obese patients. Current guidelines for antibiotic prophylaxis recommend free plasma concentrations to exceed the MIC of the antimicrobial during surgical procedure^11^. As we measured total plasma concentrations, we calculate the target concentration threshold taking into account a 80% rate of protein binding found in the literature for cefazolin in non-obese^27^ and obese patients^28^. The MIC of cefazolin for the most common bacteria responsible for SSI after clean orthopaedic surgery is acknowledged to be 4 mg/L^29,30^, equivalent to 20 mg/L with respect to total plasma concentrations. Then, we calculated the Probability Target Attainment (PTA) at skin closure and at 4 h (recommended duration for redosing). For safety evaluation, we compared cefazolin concentrations to a cutoff value of 360 mg/L, which is associated with neurotoxicity for prolonged concentrations above this threshold^31^. Concentrations up to 1000 mg/L showed no toxicity on kidneys cells in vitro^32^. Simulations were generated with Mlxplore software (version 2016R1, Lixoft). Graphs of the results were generated using R software (version 3.2.2) with the ggplot2 package (version 2.1.0).

### Institutional review board statement

The study was conducted according to the guidelines of the Declaration of Helsinki and approved by the relevant central independent ethics committee (Comité de Protection des Personnes Sud-Est1). This study is registered at the French National Agency for Medicines and Health Products Safety (ANSM; ref: 130908A-21), EudraCT (ref: 2013-000791-15) and ClinicalTrials.gov (no. NCT02252497).

### Informed consent statement

Informed consent was obtained from all subjects involved in the study.

## Results

### Patients and data sampled

One hundred patients were enrolled in this work, with a mean age of 67 years (range 24–91), a mean total body weight of 76 kg (range 48–123) and a mean CrCL according to the CKD-EPI formula of 83 mL/min/1.73 m^2^ (range 17–129). Nine patients (9%) presented renal impairment (8% with CrCL 30 to 60 mL/min/1.73 m^2^ and 1% with CrCL < 30 mL/min/1.73 m^2^). Twenty-nine patients (29%) were obese (BMI > 30 kg/m^2^). Ninety-six patients received a single dose of 2000 mg of cefazolin before surgical incision. One patient (1%) experienced SSI (deep PJI requiring prosthesis explantation at 1 year). Table 1 displays the patient characteristics. A total of 484 cefazolin concentration measurements were acquired for PK analysis. The mean time between cefazolin injection and surgical incision was 0.85 h (range 0.18–2.23). The mean surgery duration was 1.16 h (range 0.49–2.48).

**Table 1:** Baseline patient characteristics.

Patient characteristics	Number/mean (range)
Age (years)	67 (24–91)
**Sex**	
Male	51
Female	49
Total body weight (kg)	76 (48–123)
**BMI (kg/m**^**2**^**)**	
< 30	71
30–35	24
> 35	5
**CrCl (mL/min/1.73 m**^**2**^**)**	
> 60	81
30–60	8
< 30	1
**Total cefazolin dose (mg)**	
2000	96
3000	1
4000	3
Time from injection to incision (h)	0.85 (0.18–2.23)
Surgery duration (h)	1.16 (0.49–2.48)

*BMI* Body mass index, *CrCl* Creatinine clearance according to the CKD-EPI formula.

### Population PK model

A 2-compartment model best described cefazolin concentrations. We estimated interpatient variability for the following PK parameters: elimination clearance (CL), volume of the central compartment (Vc), intercompartmental clearance (Q) and volume of the peripheral compartment (Vp), with a correlation between Vc and CL. For all parameters, the random effect variance $\omega^{2}$ was a variance covariance matrix with only one extra diagonal term corresponding to the covariance between Vc and CL, and shrinkage was less than 10%. Residual variability was best described by a proportional error model.

The covariate analysis indicated that interpatient variability for parameter CL was best explained by CrCL according to the CKD-EPI formula. With regard to the other covariates investigated, no relevant interindividual variability was detected for parameters Vc, Q and Vp. Table 2 shows the PK parameter estimates for the model. Interpatient variability in CL in the final model decreased by 18% after covariate inclusion compared to the initial values estimated by the model without covariates. Inclusion of covariates resulted in a 68.51-point reduction in the BIC.

**Table 2:** Estimates of population parameters for model building.

Parameter	Estimate (% RSE)	
	Model 1	Model 2
CL (L/h) = Ɵ1 × (CrCL/80)^Ɵ2^	–	–
Ɵ1	2.87 (3.92)	2.86 (3.26)
Ɵ2	0	0.79 (10.6)
Vc (L)	4.97 (7.93)	5.2 (6.89)
Q (L/h)	10.5 (11.2)	10.9 (11.9)
Vp (L)	4.73 (3.99)	4.56 (3.07)
Ω_CL_	39 (7.57)	32 (7.46)
Ω_Vc_	59 (9.92)	57 (9)
Ω_Q_	60 (15.6)	66 (15)
Ω_Vp_	15 (31.8)	10 (71.8)
Correlation between CL and Vc	0.71 (12.6)	0.83 (5.81)
Proportional residual variance (%)	12 (5.16)	12 (4.9)
BIC	4279.77	4211.26

Model 1: model without covariates; model 2: final model including covariates.

*RSE* Relative standard error, *CL* Clearance, *Vc* Central volume of distribution, *Vp* Peripheral volume of distribution, *Q* Intercompartmental clearance, *CrCl* Creatinine clearance (mL/min) according to the CKD-EPI formula, *Ω* Random effect variance for each parameter, *BIC* Bayesian information criteria.

**Figure 1:**
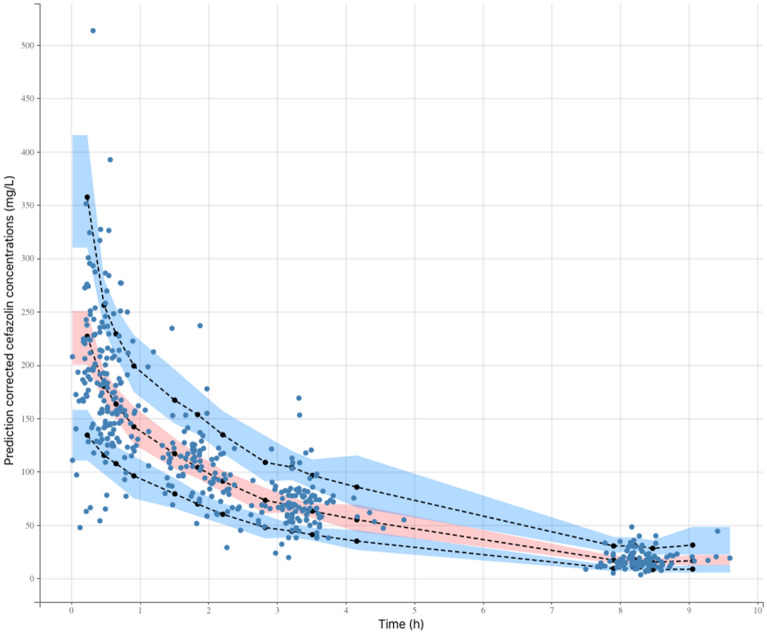
Prediction-corrected visual predictive check of the pharmacokinetic model. The 5th, 50th and 95th prediction intervals from the simulated concentrations of cefazolin are plotted against time, with the observed data superimposed. Blue and orange shaded areas are confidence interval of the prediction interval (dashed line).

**Figure 2:**
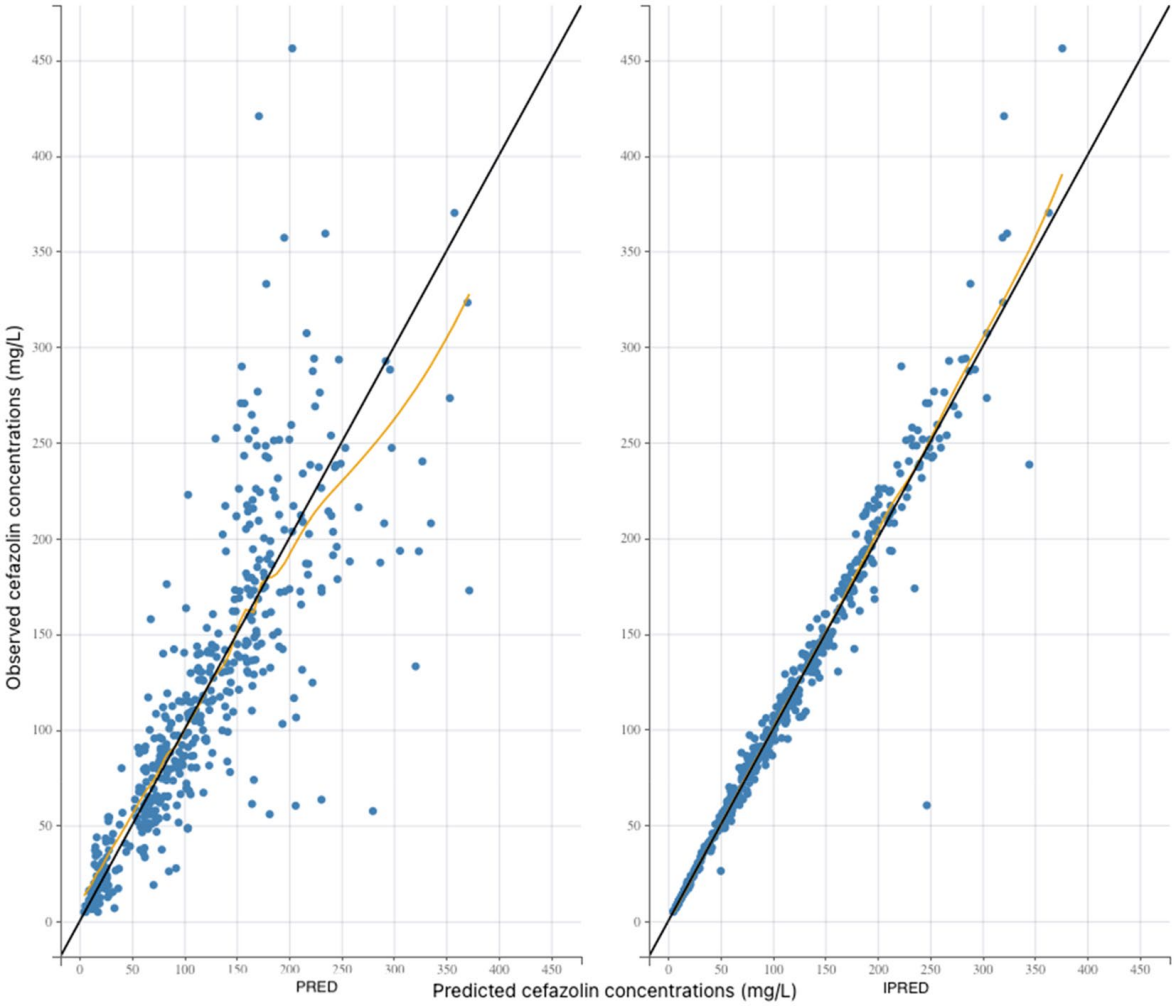
Goodness of fit plots. The black line represents the identity line. Blue circles represent the observed concentrations versus the corresponding predicted concentrations. The yellow line represents the trend line. Left panel: plot of the observed plasma concentrations (mg/L) versus population predicted plasma concentrations (PRED) (no random component). Right panel: plot of the observed plasma concentrations (mg/L) versus individual predicted plasma concentration values (IPRED) (with random component).

**Figure 3:**
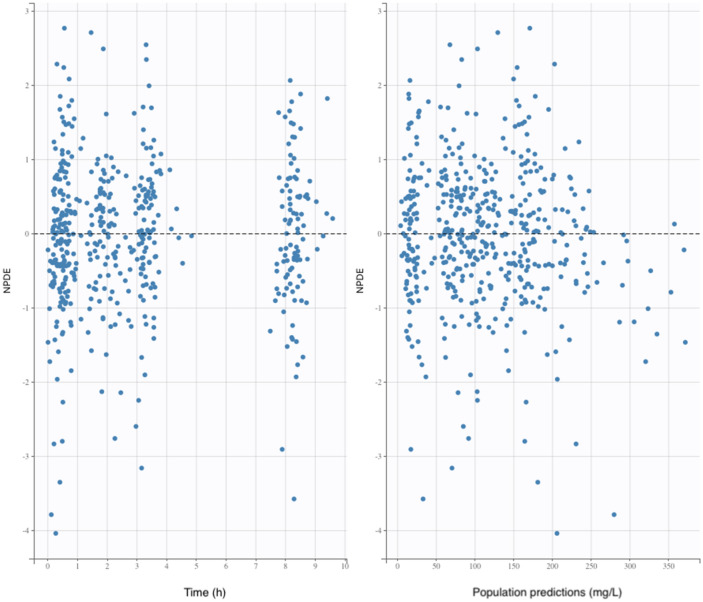
Goodness of fit plots. The black line represents the identity line. NPDE, normalized prediction distribution errors.

The pred-corrected VPC (Fig. 1) showed that the observations were easily included in the 90% prediction interval of the simulated data, confirming the good predictive performance of the model. The goodness of fit plots showed no apparent bias in model prediction (Figs. 2, 3).

### PK simulations

**Figure 4:**
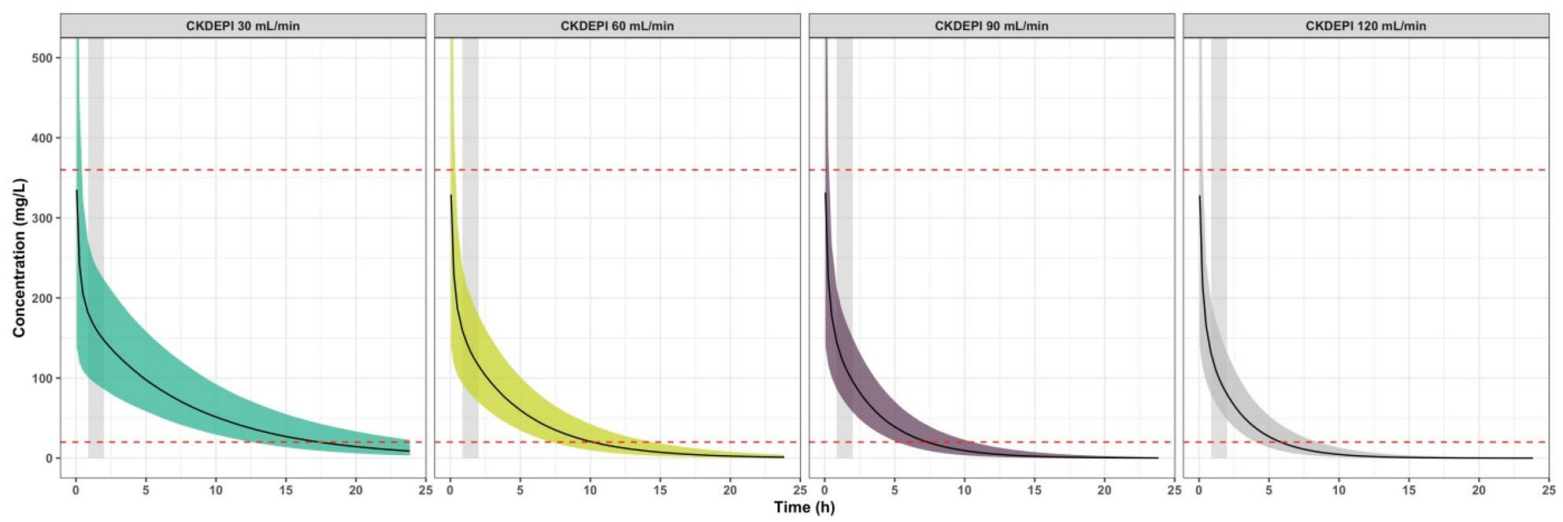
Simulations of the concentration–time course of cefazolin after a 2000 mg bolus. The simulations illustrate the effect of CrCL on cefazolin exposure over time in a patient with a CrCL of 30 mL/min/1.73 m^2^ (left panel), 60 mL/min/1.73 m^2^ (middle left panel), 90 mL/min/1.73 m^2^ (middle right panel) or 120 mL/min/1.73 m^2^ (right panel). The red dashed lines correspond to the 20 and 360 mg/litre concentration thresholds. Black solid lines correspond to the mean cefazolin concentrations. Coloured shaded areas correspond to the interpatient variability intervals estimated in our model. The grey stripe represents the surgery duration. Simulations were performed using R software (version 3.2.2) with the ggplot2 package (version 2.1.0). R Core Team (2016). R: A language and environment for statistical computing. R Foundation for Statistical Computing, Vienna, Austria. URL https://www.R-project.org/. Wickham H (2016). *ggplot2: Elegant Graphics for Data Analysis*. Springer-Verlag New York. ISBN 978–3-319–24,277-4, https://ggplot2.tidyverse.org.

**Figure 5:**
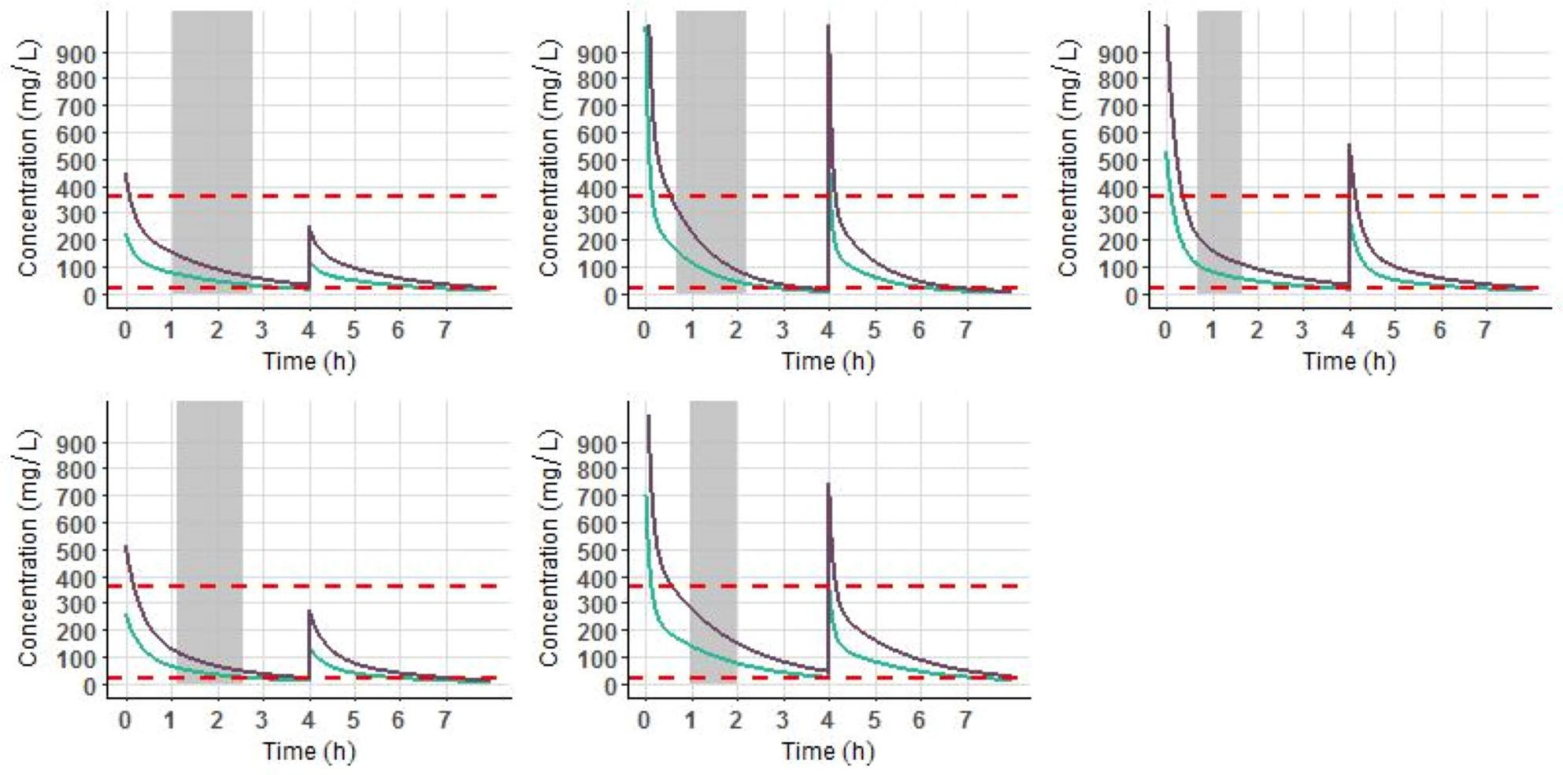
Simulations of the concentration–time course of cefazolin for the five patients included in the study with BMI > 35 kg/m^2^ and total body weight > 100 kg after a 4000 mg bolus followed by a 2000 mg at 4 h (purple line) and after a 2000 mg bolus followed by a 1000 mg at 4 h (green line). The red dashed lines correspond to the 20 and 360 mg/litre concentration thresholds. Grey stripe represents to the surgery duration. Simulations were performed using R software (version 3.2.2) with the ggplot2 package (version 2.1.0). R Core Team (2016). R: A language and environment for statistical computing. R Foundation for Statistical Computing, Vienna, Austria. URL https://www.R-project.org/. Wickham H (2016). *ggplot2: Elegant Graphics for Data Analysis*. Springer-Verlag New York. ISBN 978–3-319–24,277-4, https://ggplot2.tidyverse.org.

Simulations of the cefazolin concentration–time course after a 2000 mg single dose were investigated using the model to illustrate the effect of CrCL over time of drug exposure (Fig. 4). Table 3 presents four different CrCL profiles and the PTA to remain above the therapeutic threshold of 20 mg/litre 2.01 h (mean duration from cefazolin injection to skin closure in our study) and 4 h after cefazolin injection in each group. These results showed that the period of time above the threshold increased with renal impairment. However, regardless of renal function, cefazolin concentrations remained systemically above the threshold throughout the entire surgical period. Figure 5 displays the concentration–time course after cefazolin administration according to the French guidelines (4000 mg if BMI > 35 kg/m^2^ and total body weight > 100 kg with a 2000 mg redosing at 4 h) compared to a 2000 mg single dose with a 1000 mg redosing at 4 h. In the five patients examined, cefazolin concentrations remained above the therapeutic threshold throughout the surgical period and during 4 h with both doses. In case of surgery longer than 4 h, a 1000 mg redosing was effective to remain above the threshold. A 2000 mg redosing was associated with a longer duration spent above the toxic threshold of 360 mg/L.

**Table 3:** Estimated probability target attainment (PTA) using PK simulations with four different CrCL profiles after a 2000 mg single dose of cefazolin.

CrCl (mL/min/1.73 m^2^)	PTA at 2.01 h (%)	PTA at 4 h (%)
120	100	94.5
90	100	99.8
60	100	100
30	100	100

*CrCL* Creatinine clearance (mL/min) according to the CKD-EPI formula.

## Discussion

In this study, a population PK analysis was conducted to quantify cefazolin variability in patients undergoing THA surgery and to evaluate whether a dose adjustment to weight could optimize cefazolin exposure in obese patients.

The final model developed consisted of a 2-compartment model, where interpatient variability in clearance was best explained by creatinine clearance. During the performance of our study, another PK parametric study was performed with cefazolin in THA surgery^10^. The values of the PK parameters estimated in this study were similar to those estimated in our model. The authors also highlighted the effect of renal function on cefazolin exposure. However, there were some limitations to the previous work due to a limited sampling design. The authors of this study developed their model on the basis of sparse data, and their measurements did not extend longer than 4 h after cefazolin administration. Elimination clearance estimation was more robust in our study due to the higher number of cefazolin concentrations measured. Moreover, in our study, residual concentrations were measured until 8 h after cefazolin administration. Finally, Komatsu’s study was performed in a Japanese population. Ethnicity is known to be a factor affecting drug PK, and these results may not be applicable to a Caucasian population^33^.

Among the various body size descriptors tested in our covariate analysis, weight did not affect cefazolin concentrations. However, subjects with a large range of body weights were included in our study population, with 29% being obese patients. These results were in accordance with previous studies performed with cefazolin in THA surgery^10,34^. Cefazolin is a water-soluble antibiotic that does not penetrate adipose tissue regardless of intravenous dose, and drug disposition is not affected to a great extent by body weight, as the volume of distribution of such a hydrophilic drug remains quite stable^15^. A literature review showed that cefazolin concentrations were not affected by body weight^15^. Regarding our simulations (Fig. 5), increasing cefazolin dosing in obese patients did not improve cefazolin exposure during surgery. A 2000 mg single dose was sufficient to maintain therapeutic concentrations throughout the intraoperative period in the five obese patients simulated in our study. In case of surgery longer than 4 h, a 1000 mg redosing was efficient to remain above the threshold, a 2000 mg redosing being associated higher concentrations above the toxic threshold of 360 mg/L. Therefore, dosing guidelines concerning weight adaptation for cefazolin administration in obese patients undergoing THA surgery should be revised.

PK simulations using our final model were conducted to evaluate the influence of creatinine clearance on cefazolin concentrations (Fig. 4). Regarding these simulations, the presence of renal failure increased the duration for which cefazolin concentrations were above 20 mg/litre. However, after a 2000 mg single injection, cefazolin concentrations were systematically above the therapeutic threshold throughout surgery, regardless of CrCL. PTA was close to 100% for each profile of renal function at skin closure and at 4 h. Thus, adjusting cefazolin administration to renal function in THA does not seem to be obligatory regarding the range of concentrations observed in our study. A single 2000 mg preoperative dose without adaptation to CrCL efficiently reaches therapeutic concentrations throughout the entire surgical period. However, the presence of renal failure may lead to overdosing using this strategy. But using our simulations, the toxic threshold of 360 mg/L was never reached after a single dose. To our knowledge, no antibiotic nephrotoxicity has been demonstrated for such doses of cefazolin.

Regarding our simulations, intraoperative redosing to maintain therapeutic concentrations during surgery was not necessary. THA is a short operation, and a single 2000 mg preoperative dose efficiently reached cefazolin concentrations above the efficacy threshold during the entire intraoperative period. Therefore, it seems unnecessary to propose multiple doses to optimize cefazolin administration in THA. These results seem to be in accordance with other clinical studies, suggesting that a single dose of antibiotic prophylaxis with cefazolin is as effective as multiple doses for SSIs prevention after THA surgery^12,35^. Nevertheless, the optimal duration of cefazolin prophylaxis remains unclear. Cephalosporins are time-dependent antibiotics^36^, and a time above the MIC should be taken into consideration for optimal antimicrobial activity. It is unknown whether cefazolin concentrations should be kept above the therapeutic threshold up to 24 h to improve antibiotic prophylaxis efficacy. The results from previous studies demonstrate that a longer duration above the MIC is associated with a better outcome in patients presenting infection in intensive care^37^. However, the relationship between antibiotic prophylaxis duration and cefazolin and the SSIs rate after THA has not been established.

This work has several limitations. First, the biologic threshold for cefazolin effective prophylaxis was acknowledged to be 4 mg/L in our study, with respect to free plasma concentrations. As we measured total plasma concentrations, we took into account a 80% extent of of protein binding to calculate a new threshold of 20 mg/L with respect to total concentrations. While it is demonstrated that the optimal bactericidal activity of beta-lactam is achieved when antibiotic concentrations attain fourfold the MIC against the aimed pathogens^38,39^, it is unclear whether these concentrations should be similar for SSIs prevention and for infection treatment. Current guidelines for antibiotic prophylaxis recommend that the free plasma concentrations should exceed the MIC of the antimicrobial during surgical procedure^11^. CoNS and Staphylococcus aureus are the most encountered bacteria involved in SSI after THA surgery^8^. The MIC of Staphylococcus aureus is usually ≤ 2 mg/L and the MIC of CoNS is higher (≤ 4 mg/L)^29,30^. Thus, we chose a 4 mg/L target for effective prophylaxis. But we did not evaluate the relation between antibiotic exposure after prophylactic administration and SSI rate after surgery. In fine, the biologic threshold used in this study may be inappropriate. The relationship between cefazolin exposure and SSI should be evaluated with a PK/PD analysis.

Another limitation of this study is that we only took CoNS and methicillin-sensitive Staphylococcus aureus into consideration as the targets of cefazolin prophylaxis. However, the increasing prevalence of methicillin-resistant Staphylococcus aureus (MRSA) and cefazolin-resistant coagulase-negative staphylococci (CRCoNS) should be a cause for concern^40^. Whereas MRSA is usually not reported as a contaminant in clean orthopaedic surgery^41^, the increasing resistance of CoNS strains over the years must be taken into consideration^42^. Some authors have proposed reaching higher levels of cefazolin concentrations to target the MIC of cefazolin against CRCoNS^40^. However, the high doses necessary to reach such concentrations could lead to overdosage and toxicity, and the relationship between cefazolin concentrations and postoperative infections has not been shown. Moreover, the rate of PJI in our population remained nonsignificant (1%). Thus, it appears unnecessary to reach higher concentration thresholds to target CRCoNS.

Finally, we did not measure antibiotic concentrations at the target site. Current guidelines recommend reaching therapeutic concentrations at the location of surgery for optimal antimicrobial effects^43^. As antibiotics may not be equally distributed throughout body tissues after intravenous administration, one can consider that it is necessary for an antibiotic to reach therapeutic concentrations in the plasma and at the local target site^44^. However, a review that investigated the tissue distribution of cefazolin in orthopaedic surgery reported that there was a wide range of target site concentrations among the studies evaluated^45^. In Komatsu’s study, PK modelling at the target site was developed from 1 sample point per patient. Moreover, the volume of distribution of 36 L for the synovial capsule found in their study might be inappropriate. In fact, this volume is not identifiable and it could have been fixed to an a priori anatomical value in order to perform a better estimation. In total, it appears that Komatsu et al. were probably not in a position to report Vp estimation in an effective way. However, their results showed that cefazolin concentrations in the hip joint capsule were approximately 10% of the corresponding serum concentrations^10^. Cefazolin is a water-soluble drug with strong protein-binding characteristics, and it probably does not penetrate the hip joint capsule. However, the infection rate in our study remained insignificant. Thus, the tissue distribution of cefazolin and its implications in the prevention of postoperative infections need to be assessed in additional studies.

In conclusion, we developed a PK model of cefazolin for adult patients undergoing THA surgery. Cefazolin interindividual PK variability was partly explained by CrCL. Our PK simulations showed that a 2000 mg single injection without adaptation to weight or renal function and without intraoperative reinjection was efficient at maintaining therapeutic plasma concentrations throughout the entire surgical period. Dose adjustment to weight in obese patients did not improve cefazolin exposure. However, the optimal plasma and target site concentrations to reach and the duration at which these concentrations should be maintained remain unclear. Further research should be conducted to investigate the PK/PD relationship between cefazolin exposure and SSI after THA surgery.

## References

[1] Lindeque B, Hartman Z, Noshchenko A, Cruse M (2014). Infection after primary total hip arthroplasty. Orthopedics.

[2] Whitehouse JD, Friedman ND, Kirkland KB, Richardson WJ, Sexton DJ (2002). The impact of surgical-site infections following orthopedic surgery at a community hospital and a university hospital: Adverse quality of life, excess length of stay, and extra cost. Infect. Control Hosp. Epidemiol..

[3] Cahill JL, Shadbolt B, Scarvell JM, Smith PN (2008). Quality of life after infection in total joint replacement. J. Orthop. Surg. (Hong Kong).

[4] Vanhegan IS, Malik AK, Jayakumar P, Ul Islam S, Haddad FS (2019). A financial analysis of revision hip arthroplasty: the economic burden in relation to the national tariff. J Bone Joint Surg Br..

[5] Hill C, Flamant R, Mazas F, Evrard J (1981). Prophylactic cefazolin versus placebo in total hip replacement. Report of a multicentre double-blind randomised trial. Lancet.

[6] Hansen E, Belden K, Silibovsky R, Vogt M, Arnold WV, Bicanic G, Bini SA, Catani F, Chen J, Ghazavi MT, Godefroy KM, Holham P, Hosseinzadeh H, Kim KI, Kirketerp-Møller K, Lidgren L, Lin JH, Lonner JH, Moore CC, Papagelopoulos P, Poultsides L, Randall RL, Roslund B, Saleh K, Salmon JV, Schwarz EM, Stuyck J, Dahl AW, Yamada K (2014). Perioperative antibiotics. J. Arthroplasty.

[7] Neu HC (1984). Cephalosporin antibiotics as applied in surgery of bones and joints. Clin. Orthop. Relat. Res..

[8] Tande AJ, Patel R (2014). Prosthetic joint infection. Clin. Microbiol. Rev..

[9] Quintiliani R, Nightingale C (1984). Principles of antibiotic usage. Clin. Orthop. Relat. Res..

[10] Komatsu T, Natsume Y, Uchiyama K, Ikeda S, Tomoda Y, Takayama Y, Takaso M, Hanaki H, Atsuda K (2021). Population pharmacokinetic and pharmacodynamic target attainment analysis of cefazolin in the serum and hip joint capsule of patients undergoing total hip arthroplasty. Antimicrob. Agents Chemother..

[11] Bratzler, D. W., Dellinger, E. P., Olsen, K. M., Perl, T. M., Auwaerter, P. G., Bolon, M. K., Fish, D. N., Napolitano, L. M., Sawyer, R. G., Slain, D., Steinberg, J. P., Weinstein, R. A.; American Society of Health-System Pharmacists; Infectious Disease Society of America; Surgical Infection Society; Society for Healthcare Epidemiology of America. Clinical practice guidelines for antimicrobial prophylaxis in surgery. *Am. J. Health Syst. Pharm.* **70**(3), 195–283. 10.2146/ajhp120568 (2013). 10.2146/ajhp120568 23327981

[12] Tang WM, Chiu KY, Ng TP, Yau WP, Ching PT, Seto WH (2003). Efficacy of a single dose of cefazolin as a prophylactic antibiotic in primary arthroplasty. J. Arthroplasty.

[13] Parvizi J, Gehrke T, Chen AF (2013). Proceedings of the international consensus on periprosthetic joint infection. Bone Joint J..

[14] Anderson DJ, Podgorny K, Berríos-Torres SI, Bratzler DW, Dellinger EP, Greene L, Nyquist AC, Saiman L, Yokoe DS, Maragakis LL, Kaye KS (2014). Strategies to prevent surgical site infections in acute care hospitals: 2014 update. Infect. Control Hosp. Epidemiol..

[15] Blum S, Cunha CB, Cunha BA (2019). Lack of pharmacokinetic basis of weight-based dosing and intra-operative re-dosing with cefazolin surgical prophylaxis in obese patients: Implications for antibiotic stewardship. Surg. Infect. (Larchmt.).

[16] Hussain Z, Curtain C, Mirkazemi C, Gadd K, Peterson GM, Zaidi STR (2019). Prophylactic cefazolin dosing and surgical site infections: Does the dose matter in obese patients?. Obes. Surg..

[17] Zufferey, P. J., Lanoiselée, J., Chapelle, C., Borisov, D. B., Bien, J. Y., Lambert, P., Philippot, R., Molliex, S., Delavenne, X.; Investigators of the PeriOpeRative Tranexamic Acid in Hip Arthroplasty (PORTO) Study. Intravenous tranexamic acid bolus plus infusion is not more effective than a single bolus in primary hip arthroplasty: A randomized controlled trial. *Anesthesiology*. **127**(3), 413–422. 10.1097/ALN.0000000000001787 (2017). 10.1097/ALN.0000000000001787 28692467

[18] Martin, C., Auboyer, C., Boisson, M., Dupont, H., Gauzit, R., Kitzis, M., Leone, M., Lepape, A., Mimoz, O., Montravers, P., Pourriat. J. L.; Steering committee of the French Society of Anaesthesia and Intensive Care Medicine (SFAR) responsible for the establishment of the guidelines. Antibioprophylaxis in surgery and interventional medicine (adult patients). Update 2017. *Anaesth. Crit. Care Pain Med.* **38**(5), 549–562. 10.1016/j.accpm.2019.02.017 (2019). 10.1016/j.accpm.2019.02.017 30836191

[19] Lanoiselée J, Zufferey PJ, Hodin S, Tamisier N, Gergelé L, Palao JC, Campisi S, Molliex S, Morel J, Delavenne X, Ollier E (2020). Pharmacokinetic model for cefuroxime dosing during cardiac surgery under cardiopulmonary bypass. Antimicrob. Agents Chemother..

[20] Lanoiselée J, Zufferey PJ, Ollier E, Hodin S, Delavenne X; PeriOpeRative Tranexamic Acid in Hip Arthroplasty (PORTO) Study Investigators. Is tranexamic acid exposure related to blood loss in hip arthroplasty? A pharmacokinetic-pharmacodynamic study. *Br. J. Clin. Pharmacol.* **84**(2), 310–319. 10.1111/bcp.13460 (2018). 10.1111/bcp.13460 PMC5777664 29193211

[21] Delyon B, Lavielle M, Moulines E (1999). Convergence of a stochastic approximation version of the EM algorithm. Ann. Stat..

[22] Cockcroft DW, Gault MH (1976). Prediction of creatinine clearance from serum creatinine. Nephron.

[23] Levey, A. S., Stevens, L. A., Schmid, C. H., Zhang, Y. L., Castro, A. F. 3rd, Feldman, H.I., Kusek, J. W., Eggers, P., Van Lente, F., Greene, T., Coresh, J.; CKD-EPI (Chronic Kidney Disease Epidemiology Collaboration). A new equation to estimate glomerular filtration rate. *Ann. Intern. Med.* **150**(9), 604–612. 10.7326/0003-4819-150-9-200905050-00006 (2009) (**Erratum in: Ann Intern Med. 2011;155(6):408**). 10.7326/0003-4819-150-9-200905050-00006 PMC2763564 19414839

[24] Bergstrand M, Hooker AC, Wallin JE, Karlsson MO (2011). Prediction-corrected visual predictive checks for diagnosing nonlinear mixed-effects models. AAPS J..

[25] Brendel K, Comets E, Laffont C, Mentré F (2010). Evaluation of different tests based on observations for external model evaluation of population analyses. J. Pharmacokinet. Pharmacodyn..

[26] Delattre M, Lavielle M, Poursat MA (2014). A note on BIC in mixed-effects models. Electron. J. Stat..

[27] Kamani G, Low CL, Valerie TTH, Chui WK (1998). HPLC determination of cefazolin in plasma, urine and dialysis fluid. J. Pharm. Pharmacol..

[28] van Kralingen S, Taks M, Diepstraten J, van de Garde EM, van Dongen EP, Wiezer MJ, van Ramshorst B, Vlaminckx B, Deneer VH, Knibbe CA (2011). Pharmacokinetics and protein binding of cefazolin in morbidly obese patients. Eur. J. Clin. Pharmacol..

[29] EUCAST. Antimicrobial wild type distributions of microorganisms. https://mic.eucast.org/search/?search%5Bmethod%5D=mic&search%5Bantibiotic%5D=27&search%5Bspecies%5D=-1&search%5Bdisk_content%5D=-1&search%5Blimit%5D=50. Accessed 6 Aug 2021.

[30] Grégoire M, Dumont R, Ronchi L, Woillard JB, Atthar V, Letessier E, Cinotti R, Roquilly A, Deslandes G, Jolliet P, Asehnoune K, Dailly E (2018). Prophylactic cefazolin concentrations in morbidly obese patients undergoing sleeve gastrectomy: Do we achieve targets?. Int. J. Antimicrob. Agents.

[31] Moore TD, Bechtel TP, Ayers LW (1981). Effect of multidose therapy on cerebrospinal fluid penetration of cefazolin. Am. J. Hosp. Pharm..

[32] Bruinsma BG, Post IC, van Rijssen LB, de Boer L, Heger M, Zaat SA, van Gulik TM (2013). Antibiotic prophylaxis in (sub)normothermic organ preservation: in vitro efficacy and toxicity of cephalosporins. Transplantation.

[33] Yasuda SU, Zhang L, Huang SM (2008). The role of ethnicity in variability in response to drugs: Focus on clinical pharmacology studies. Clin. Pharmacol. Ther..

[34] Sharareh B, Sutherland C, Pourmand D, Molina N, Nicolau DP, Schwarzkopf R (2016). Effect of body weight on cefazolin and vancomycin trabecular bone concentrations in patients undergoing total joint arthroplasty. Surg. Infect. (Larchmt.).

[35] Veltman ES, Lenguerrand E, Moojen DJF, Whitehouse MR, Nelissen RGHH, Blom AW, Poolman RW (2020). Similar risk of complete revision for infection with single-dose versus multiple-dose antibiotic prophylaxis in primary arthroplasty of the hip and knee: Results of an observational cohort study in the Dutch Arthroplasty Register in 242,179 patients. Acta Orthop..

[36] Jacobs MR (2001). Optimisation of antimicrobial therapy using pharmacokinetic and pharmacodynamic parameters. Clin. Microbiol. Infect..

[37] Roberts. J. A., Paul, S. K., Akova, M., Bassetti, M., De Waele, J. J., Dimopoulos, G., Kaukonen, K. M., Koulenti, D., Martin, C., Montravers, P., Rello, J., Rhodes, A., Starr, T., Wallis, S. C., Lipman, J.; DALI Study. DALI: Defining antibiotic levels in intensive care unit patients: are current β-lactam antibiotic doses sufficient for critically ill patients? Clin. Infect. Dis. **58**(8), 1072–1083. 10.1093/cid/ciu027 (2014). 10.1093/cid/ciu027 24429437

[38] Craig WA, Ebert SC (1992). Continuous infusion of beta-lactam antibiotics. Antimicrob. Agents Chemother..

[39] Bundtzen RW, Gerber AU, Cohn DL, Craig WA (1981). Postantibiotic suppression of bacterial growth. Rev. Infect. Dis..

[40] Yamada K, Matsumoto K, Tokimura F, Okazaki H, Tanaka S (2011). Are bone and serum cefazolin concentrations adequate for antimicrobial prophylaxis?. Clin. Orthop. Relat. Res..

[41] Bernard L, Sadowski C, Monin D, Stern R, Wyssa B, Rohner P, Lew D, Hoffmeyer P (2004). The value of bacterial culture during clean orthopedic surgery: A prospective study of 1,036 patients. Infect. Control Hosp. Epidemiol..

[42] Rafiq I, Gambhir AK, Wroblewski BM, Kay PR (2006). The microbiology of infected hip arthroplasty. Int. Orthop..

[43] Kirby JP, Mazuski JE (2009). Prevention of surgical site infection. Surg. Clin. N. Am..

[44] Müller M, dela Peña A, Derendorf H (2004). Issues in pharmacokinetics and pharmacodynamics of anti-infective agents: Distribution in tissue. Antimicrob. Agents Chemother..

[45] Sanders FRK, Goslings JC, Mathôt RAA, Schepers T (2019). Target site antibiotic concentrations in orthopedic/trauma extremity surgery: Is prophylactic cefazolin adequately dosed? A systematic review and meta-analysis. Acta Orthop..

